# The Association of Weight Reduction and Other Variables after Bariatric Surgery with the Likelihood of SARS-CoV-2 Infection

**DOI:** 10.3390/jcm12124054

**Published:** 2023-06-15

**Authors:** Noam Frish, Ariel Israel, Shai Ashkenazi, Shlomo Vinker, Ilan Green, Avivit Golan-Cohen, Eugene Merzon

**Affiliations:** 1 Adelson School of Medicine, Ariel University, Ariel 40700, Israel; noamfrish12@gmail.com (N.F.); shaias@ariel.ac.il (S.A.); 2 Leumit Health Services, Tel Aviv 64738, Israel; aisrael@leumit.co.il (A.I.); svinker@leumit.co.il (S.V.); igreen@leumit.co.il (I.G.); agolanchoen@leumit.co.il (A.G.-C.); 3 Sackler Faculty of Medicine, Tel Aviv University, Tel Aviv 69978, Israel

**Keywords:** obesity, overweight, bariatric surgery, weight loss, COVID-19, pandemic, corona virus, SARS-CoV-2, population study

## Abstract

Background and aims: Although obesity has been confirmed as a risk factor for SARS-CoV-2 infection and its severity, the role of post-bariatric surgery (BS) variables and the infection is unclear. We, therefore, aimed to study comprehensively the relationship between the extent of weight reduction after surgery and other demographic, clinical, and laboratory variables with the rates of SARS-CoV-2 infection. Methods: A population-based cross-sectional study was performed, utilizing advanced tracking methodologies on the computerized database of a nation-wide health maintenance organization (HMO). The study population included all HMO members aged ≥18 years that had been tested at least once for SARS-CoV-2 during the study period and underwent BS at least one year before their testing. Results: Of the total 3038 individuals who underwent BS, 2697 (88.78%) were positive for SARS-CoV-2 infection and 341 (11.22%) were negative. Multivariate regression analysis demonstrated that the body mass index and the amount of weight reduction after the BS were not related to the likelihood of SARS-CoV-2 infection. Post-operative low socioeconomic status (SES) and vitamin D3 deficiency were associated with significant and independent increased rates of SARS-CoV-2 infection (odds ratio [OR] 1.56, 95% confidence interval [CI], 1.19–2.03, *p* < 0.001; and OR 1.55, 95% CI, 1.18–2.02, *p* < 0.001; respectively). Post-operative physical activity > 3 times/week was associated with a significant and independent reduced rate of SARS-CoV-2 infection (OR 0.51, 95% CI, 0.35–0.73, *p* < 0.001). Conclusion: Post-BS vitamin D3 deficiency, SES, and physical activity, but not the amount of weight reduction, were significantly associated with the rates of SARS-CoV-2 infection. Healthcare workers should be aware of these associations after BS and intervene accordingly.

## 1. Introduction

### 1.1. COVID-19 Pandemic

The coronavirus disease 2019 (COVID-19) pandemic, caused by the severe acute respiratory syndrome coronavirus 2 (SARS-CoV-2), impacted enormously global health; up to 28 February 2022, the World Health Organization (WHO) has recorded more than 758 million confirmed cases, of whom ~6.9 million were fatal [1]. The true number of fatalities is several-fold higher as documented by a comprehensive measurement of excessive deaths [2]. More than three years into the pandemic, it is obvious that the rate of SARS-CoV-2 infection and its severity have a major inter-individual variability, ranging from asymptomatic infection in nearly 50% of the infected individuals to a severe course with multi-organ involvement, short- and long-term complications and deaths [3,4]. Furthermore, the enormous hospitalization rates associated with the COVID-19 pandemic affected overall hospital activities and the perceived quality of care [5,6,7]. It has been demonstrated that several demographic variables [3,8,9,10] and underlying cardiac, respiratory, and other medical conditions [5,11,12,13,14,15,16,17,18] affect the susceptibility to SARS-CoV-2 infection and its severity. On the other hand, COVID-19 has a considerable impact on the cardiovascular and respiratory systems, including pulmonary hypertension [2,19,20,21].

### 1.2. Obesity and SARS-CoV-2 Infection

Several studies, using distinct methodologies, have documented that obesity predisposes to a severe complicated course [11,17,22] and fatal outcome [22,23,24,25] of SARS-CoV-2 infection. A systematic review of 45,650 individuals from 33 studies showed that those with a high body mass index (BMI)-defined obesity had significantly higher Odds Ratios (ORs) for hospitalization, admission to intensive care units, requirement of invasive ventilation, and death [11]. Moreover, the severity of COVID-19 appears to rise with increasing BMI [22]; overweight was also associated with its complications [22] and fatality [23]. It was likewise confirmed by a survey of 444,649 adults randomly selected from the database of the United States Behavioral Risk Factor Surveillance System [17].

The strong association between overweight/obesity and the severity of SARS-CoV-2 infection has biological and pathophysiological plausibility. This includes chronic pro-inflammatory state with increased serum levels of interleukin 6 (IL-6), tumor necrosis factor α (TNF-α) and C-reactive protein, excessive oxidative stress response, and impaired immunity with altered natural killer cell polarization that are commonly observed in individuals with increased BMI and accelerate the deleterious downstream immunologic effects of SARS-CoV-2 [22,26,27]. Obesity is associated, in addition, with reduced lung functions, hypertension, cardiovascular dysfunction, vascular damage, and reduced renal function, factors related to negative clinical outcomes of SARS-CoV-2 infection [22,28].

### 1.3. Bariatric Surgery

Bariatric surgery is increasingly used as a very effective therapeutic option for individuals with severe obesity [28,29]. Comprehensive reviews [27,28] and a meta-analysis of 164 studies [29] showed that bariatric surgery decreases the long-term risk of all-cause mortality compared with matched non-surgical patients. The surgery improved obesity-related comorbidities such as cardiovascular disease, hypertension, diabetes mellitus, obstructive sleep apnea, dyslipidemia, and inflammatory markers [28,29]. Among diabetic patients, at least 12 randomized controlled trials comparing bariatric surgery with conventional therapeutic strategies (i.e., lifestyle change and medications) showed that surgery was superior to controlling hyperglycemia, reducing cardiovascular and overall mortality risk, improving the quality of life, and reducing renal complications [27,28,29]. It should be remembered, however, that bariatric surgery-related weight loss can be associated with non-surgical complications, including malabsorption, nutrient deficiencies, and hormonal disturbances [30,31].

### 1.4. Bariatric Surgery and SARS-CoV-2 Infection

The relations between bariatric surgery and SARS-CoV-2 infection and vice versa are complex and have not been fully elucidated yet. On one hand, COVID-19 and its related behavioral changes reduced the effectiveness of bariatric surgery in terms of weight loss [32] and persistent type-2 diabetes [32,33]. On the other hand, studies on the influence of previous bariatric surgery on the severity of COVID-19 showed limited and conflicting results. A comparative study showed that patients who had COVID-19 before or within 3 months after bariatric surgery had a similar severity [34], while a telephone questionnaire yielded similar rates of COVID-19 in patients who underwent bariatric surgery compared to candidates, with a shorter duration of symptoms in the operated group [35]. A retrospective study showed that probable COVID-19 was less severe in operated patients [32], as did a study on selected patients with underlying diabetes who underwent surgery [36]. To our knowledge, no population-based studies that have examined all confirmed SARS-CoV-2 infections after bariatric surgery have been reported, nor have the associations between post-bariatric surgery laboratory data and the infection.

### 1.5. Aim of Study

Our study aimed to provide a comprehensive understanding of the association between bariatric surgery and the likelihood of SARS-CoV-2 infection. By analyzing a large-scale population-based database, we investigated the association between post-surgery weight reduction and infection rates while considering various demographic, clinical, and laboratory variables. We aimed to uncover valuable insights into SARS-CoV-2 infection among post-bariatric surgery patients and shed light on broader implications for the needed follow-up.

## 2. Materials and Methods

### 2.1. Study Design

We conducted a population-based cross-sectional study utilizing information from the Leumit Health Services (LHS) database, a large nation-wide health maintenance organization in Israel, which provided services to about 725,000 members during the study period. LHS has a comprehensive computerized database, continuously updated concerning demographics, medical visits, diagnoses, laboratory tests, hospitalizations, and medication prescriptions of the registered subjects. All LHS members hold equal general health insurance and similar access to health services. Diagnoses are entered and updated according to the International Classification of Diseases 9th revision (ICD-9). The validity of the registry has been previously examined and confirmed as high [37]. The study protocol was approved by the statutory clinical ethics committees of LHS and the Shamir Medical Center Institutional Review Board on Human Research (0129-20-LEU).

### 2.2. Study Period and Population

The study period was from 1 February 2020 to 31 December 2020. The study population included all LHS members aged ≥18 years that had been tested at least once for SARS-CoV-2 during the study period and underwent bariatric surgery at least one year prior to their SARS-CoV-2 testing. Data of each subject were collected from the LHS computerized database using advanced tracking methodologies and included age, gender, socioeconomic status (SES), weight, height, BMI, smoking status, comorbidities, and laboratory data, including the results of SARS-CoV-2 testing. The COVID-19 vaccine was not available in our country before or during the study period.

Three types of bariatric surgery were identified based on the relevant International Classification of Diseases, Ninth Revision (ICD-9) code in the Leumit Health Service database—sleeve gastrectomy, gastric bypass, and laparoscopic banding.

According to Israeli Ministry of Health criteria during the study period, testing for SARS-CoV-2 infection was performed only upon physician referral for testing, which was based on direct exposure to a confirmed COVID-19 patient and/or presentation of symptoms suggesting COVID-19. Nasopharyngeal swabs were taken and examined for SARS-CoV-2 by real-time RT-PCR performed with internal positive and negative controls, according to WHO guidelines. The Allplex 2019-nCoV assay (Seegene, Seoul, Korea) was used until 10 March 2020, and thereafter—the COBAS SARS-CoV-2 6800/8800 assay (Roche Pharmaceuticals, Basel, Switzerland) was used.

### 2.3. Definitions

Levels of SES was defined according to the Israeli Central Bureau of Statistics classification to 20 levels; levels one to nine were designated as low SES, and 10 to 20 as medium-high SES. Frequency of physical activity (PA) was routinely recorded during family physician visits according to the patient report and categorized into three categories: no PA, PA 1–3 times per week, and PA more than 3 times per week. BMI was calculated as body weight (in kilograms) divided by the square of the body height (in meters) and expressed in units of kg/m^2^. Poorly controlled diabetes mellitus was defined as hemoglobin A1C (Hb A1C) ≥ 7%. Serum vitamin D3 levels below 20 ng/mL and vitamin B12 levels below 200 pg/mL were defined as respective deficiencies.

### 2.4. Statistical Analysis

Statistical analyses were performed using the STATA 12 software (StataCorp LP, College Station, TX, USA). All tests were two-sided with a predefined α of <0.05. The SARS-CoV-2 positive and negative groups of the patients after bariatric surgery were compared regarding many characteristics. The primary outcome was the probability of having SARS-CoV-2 infection, examined as related to multiple demographic, clinical, and laboratory variables, including the degree of weight reduction after the bariatric surgery. Differences between the two groups were assessed by the Fisher exact test for categorical variables, and the two-tailed Wilcoxon Mann–Whitney U for continuous variables. Categorical data were expressed as rates and continuous variables with normal distribution as mean ± standard deviation (SD). Stratified analyses included an initial univariate evaluation of risk estimates for SARS-CoV-2 positivity, with a subsequent multivariable regression model to examine adjusted odds ratios (ORs), 95% confidence interval [CI], and significance (p) of independent associations with SARS-CoV-2 infection in patients after bariatric surgery. The Benjamini-Hochberg procedure was utilized to control the false discovery rate for multiple testing.

## 3. Results

### 3.1. Study Population

Of the 724,129 individuals registered in LHS, a total of 3038 patients who underwent bariatric surgery and SARS-CoV-2 RT PCR testing were identified and defined as the study population. None of our participants had SARS-CoV-2 infection before the beginning of the study period, nor had anyone been vaccinated against COVID-19 before or during the study period. Of the study group, 341 (11.22%) tested positive for SARS-CoV-2, and 2697 (88.78%) tested negative. These two groups were compared regarding variables that might be affected by bariatric surgery (such as weight reduction and laboratory data) and variables that have been associated with SARS-CoV-2 infection. There were no statistically significant differences in the rates of SARS-CoV-2 infection by the type of bariatric surgery. The rates of SARS-CoV-2-positive vs SARS-CoV-2-negative individuals in the various type of bariatric surgery were sleeve gastrectomy [142 (42.14%) vs. 1140 (42.24%)], gastric bypass [97 (28.73%) vs. 776 (28.87%)], and laparoscopic banding [69 (19.71%) vs. 530 (19.62%)].

### 3.2. Demographic Characteristics of the Study Groups

Table 1 shows the demographic characteristics of the patients after bariatric surgery with positive and negative SARS-CoV-2 tests. Age (both mean and by categories) and gender did not show significant differences between the two groups. Low SES compared to middle-high SES had a significant positive association with SARS-CoV-2 infection (69.5% vs. 59.9%), while smoking compared to no smoking had a significant negative association (12.1% vs. 22.9%), with *p* < 0.001 for both. Additional details are shown in Table 1.

### 3.3. Clinical and Laboratory Variables of the Study Groups

Table 2 compares multiple clinical and laboratory variables between individuals after bariatric surgery who had positive SARS-CoV-2 tests with those who had negative SARS-CoV-2 tests. BMI, both before and after the surgery, was very similar in the two groups; likewise, the weight change after the bariatric surgery and the levels of change did not show significant differences between the groups. Rates of diabetes and hypertension showed no significant differences between the two groups, as did many laboratory data (details in Table 2). The only significant difference was in the post-bariatric surgery vitamin D3 serum level: 17.19 ± 8.55 and 19.09 ± 9.16 ng/mL in the SARS-CoV-2 positive and negative groups, respectively (*p* < 0.001). 

### 3.4. Multivariate Analysis

Variables that were significant in the univariate examination and those known as related to SARS-CoV-2 infection were entered into the multivariate regression model to determine the adjusted ORs and CIs for SARS-CoV-2 infection in patients after bariatric surgery. As depicted in Table 3, age categories and gender were not significantly associated with infection rates, nor did the amount of weight loss after the bariatric surgery. Low SES and post-operative vitamin D3 deficiency were associated with an increased likelihood of SARS-CoV-2 infection (OR 1.56, 95% CI 1.19–2.03, *p* < 0.001 and OR 1.55, 95% CI 1.18–2.02, *p* < 0.001; respectively). Self-reported current smoking status and self-reported intensive level physical activity (>3 times a week) were associated with a decreased likelihood of SARS-CoV-2 infection (OR 0.51, 95% CI 0.35–0.73, *p* < 0.001 and OR 0.47, 95% CI 0.23–0.95, *p* = 0.036, respectively). Additional details are shown in Table 3.

## 4. Discussion

### 4.1. New Findings

This study presents several new findings regarding the rates of SARS-CoV-2 infections in patients after bariatric surgery.

1. Negative findings that the BMI and the amount of weight reduction after bariatric surgery were not significantly associated with the likelihood of SARS-CoV-2 infection.
2. Post-operative low SES and vitamin D3 deficiency were associated with significant and independent increased rates of SARS-CoV-2 infection.
3. Post-operative intense physical activity and smoking were associated with significantly reduced rates of SARS-CoV-2 infection.

### 4.2. Discussion of the New Findings

As obesity has been documented by multiple studies as a risk factor for SARS-CoV-2 infection and its complications [11,17,22,25], we have anticipated that low BMI and high-level weight reduction after bariatric surgery would have been associated with reduced rates of SARS-CoV-2 infection. This assumption was clearly rejected by the results of the present study. The finding is in fact in line with previous results demonstrating that intensive weight loss after bariatric surgery may lead to malabsorption [28,30,31], which has been shown as a risk factor for SARS-CoV-2 infection [38]. Bariatric surgery might also lead to nutrient and vitamin deficiencies [28,30,38] and hormonal disturbances [31] that can also affect the susceptibility to SARS-CoV-2 infection [13]. Moreover, it is plausible that the behavioral changes during the initial phases of the COVID-19 pandemic—in-home quarantines and social distancing [1,2]—affected eating habits and increased the risks of malabsorption, nutrient deficiencies, and vitamin D3 deficiency of some individuals after bariatric surgery, and thus the susceptibility to SARS-CoV-2 infection [13,39,40].

In the present study, vitamin D3 serum levels were significantly lower in patients after bariatric surgery with SARS-CoV-2 infection than in those without infection, and vitamin D3 deficiency had a significant odds ratio of 1.55 for the infection in this population. This finding is in concert with the previous observation that bariatric surgery might lead to reduced vitamin D3 levels [30,39] and with our and others’ observation that low vitamin D3 serum levels also increased the risk of SARS-CoV-2 infection in the general population [13,41,42]. It has been indeed shown that vitamin D3 has immunomodulatory effects by increasing innate immune responses, reducing the production of pro-inflammatory cytokines, and reducing survival and replication of viruses by secretion of antiviral peptides [43]. The practical implication is that physicians should pay special attention to vitamin D3 levels after bariatric surgery and, when needed, subscribe vitamin D3 supplementation.

Low socioeconomic status after bariatric surgery was also associated independently and significantly with increased susceptibility to SARS-CoV-2 infection, in agreement with previous publications of this risk factor in the general population [3,8,10,44]. We found that physical activity after bariatric surgery of at least three times a week was associated with a significantly reduced odd of SARS-CoV-2 infection, as observed in the general population regarding the rates [45] and severity [46,47] of SARS-CoV-2 infection. In particular, patients with COVID-19 who consistently met the physical activity guidelines had a significantly lower risk of hospitalization (OR 2.26, 95% CI 1.81–2.83), admission to the intensive care unit (OR 1.73, 95% CI 1.18–2.55) and death (OR 2.49, 95% CI 1.33–4.67) due to COVID-19 than patients who were consistently inactive [46]. Physicians should, therefore, be aware of the importance of physical activity after bariatric surgery. Smoking was negatively associated with the rates of SARS-CoV-2 infection, as has been previously observed in our study focused on patients with asthma [48] Other epidemiological studies on COVID-19, from Israel [49], Italia [50,51], China [52], and the United States [53] also have reported lower prevalence of smoking among SARS-CoV-2 positive individuals. This is probably not an independent factor, but secondary to behavior (such as being in open spaces outside closed buildings for smoking) and other characteristics that were not included in the study analysis. In addition, nicotine, the main addictive compound found in tobacco, has demonstrated anti-inflammatory properties across various cell types. These properties may interfere with SARS-CoV-2 infection, by involving mechanisms related to inflammation [54,55,56].

### 4.3. Strengths and Limitations

The main strength of the present study is that it is large-scale, population-based, and conducted on real-world data. An additional strength is the use of advanced digital systems to analyze a multitude of demographic, clinical, and laboratory variables that might affect the risk of SARS-CoV-2 infection after bariatric surgery. To our knowledge, this is the first study that examines comprehensively the relationship between multiple post-bariatric surgery variables and the likelihood of SARS-CoV-2 infection in a large cohort of a nation-wide database.

The major limitation of the study relates to its observational and retrospective nature with the possibility that some unrecognized biases could have affected the results. We, therefore, describe our findings with the term association and likelihood, not causality. As our population was mainly Caucasians, the findings may not be automatically generalized to other ethnic groups. It is anticipated that the present study will stimulate further research to repeat our findings in other populations and perform prospective studies to determine causality and the mechanisms involved.

## 5. Conclusions

The present population-based nation-wide study demonstrated that the BMI and the amount of weight reduction after bariatric surgery were not related to the likelihood of SARS-CoV-2 infection. Post-operative low SES and vitamin D3 deficiency were associated with significant and independent increased rates of SARS-CoV-2 infection, while post-operative intense physical activity was associated with a significant and independent reduced rate. Physicians and other healthcare workers should be aware of these associations after bariatric surgery and intervene as required. Additional research is needed to clarify the factors that influence the connection between smoking and SARS-CoV-2 infection. Given the study’s limitations, it is important to be cautious when interpreting these findings, as they should be regarded merely as an initial report intended to inspire further investigations in both basic and clinical research.

## Figures and Tables

**Table 1:** Demographic characteristics of the patients after bariatric surgery with positive and negative SARS-CoV-2 RT PCR tests.

Variables		SARS-CoV-2 Positive N = 341 (11.22%)	SARS-CoV-2 Negative N = 2697 (88.78%)	*p*-Value
Age (years), mean ± SD		40.55 ± 10.27	41.176 ± 11.10	0.323
Age category	≤20	7 (2.05%)	76 (2.82%)	0.638
	21–40	154 (45.16%)	1150 (42.64%)	
	41–60	170 (49.85%)	1370 (50.80%)	
	≥61	10 (2.93%)	101 (3.74%)	
Female Gender		249(73.02%)	1927 (71.45%)	0.544
Socioeconomic status	Low	237 (69.50%)	1616 (59.92%)	<0.001
	Middle-High	104 (30.50%)	1081 (40.08%)	
Smoking	No	292 (85.88%)	1967 (74.93%)	<0.001
	Current	41 (12.06%)	600 (22.86%)	
	Past	7 (2.06%)	58 (2.21%)	
Types of bariatric surgery	Sleeve gastrectomy	142 (42.14%)	1140 (42.24%)	0.943
	Gastric bypass	97 (28.73%)	776 (28.87%)	
	Laparoscopic banding	69 (19.71%)	530 (19.62%)	
	Missing data	33 (9.42%)	251 (9.27%)	
Intensity of physical activity	No	120 (35.40%)	1082 (41.58%)	0.101
	Occasionally	119 (35.10%)	862 (33.13%)	
	1–3 times a week	76 (22.42%)	470 (18.06%)	
	>3 times a week	24 (7.08%)	188 (7.23%)	

**Table 2:** Clinical and laboratory variables of the subjects after bariatric surgery with positive and negative SARS-CoV-2 RT PCR tests.

Variables		SARS-CoV-2 Positive N = 341 (11.22%)	SARS-CoV-2 Negative N = 2697 (88.78%)	*p*-Value
BMI (kg/m^2^) before surgery		42.95 ± 4.70	42.61 ± 4.58	0.195
BMI (kg/m^2^) after surgery		34.75 ± 7.35	34.20 ± 7.18	0.180
Weight change after surgery by category	Gain/no change	28 (8.21%)	244 (8.31%)	0.992
	Loose < 50 kg	288 (84.46%)	2280 (84.54%)	
	Loose ≥ 50 kg	25 (7.33%)	193 (7.16%)	
Diabetes mellitus		97 (28.45%)	776 (28.77%)	0.900
Hypertension		123 (36.07%)	984 (35.15%)	0.738
Hemoglobin (g%)		12.94 ± 1.78	13.00 ± 1.77	0.579
Iron level (μg/L)		71.31 ± 31.10	73.12 ± 33.54	0.343
Total cholesterol (mg/dL)		191.88 ± 43.17	191.21 ± 41.63	0.781
LDL cholesterol (mg/dL)		117.10 ± 36.13	115.45 ± 32.97	0.390
HDL cholesterol level (mg/dL)		52.12 ± 12.27	51.66 ± 12.92	0.534
Triglycerides (mg/dL)		114.53 ± 59.45	120.98 ± 86.26	0.179
Hemoglobin A1C (%)		5.68 ± 1.17	5.62 ± 0.93	0.276
TSH (mU/L)		2.10 ± 1.67	2.23 ± 3.64	0.496
Creatinine (mg/dL)		1.29 ± 8.09	1.17 ± 8.90	0.811
Urea (mg/dL)		28.20 ± 10.22	28.65 ± 10.85	0.465
Uric Acid (mg/dL)		5.20 ± 1.55	5.17 ± 1.49	0.754
Folic Acid (ng/mL)		8.90 ± 5.26	8.43 ± 5.19	0.116
Vitamin D3 (ng/mL)		17.19 ± 8.55	19.09 ± 9.16	<0.001
Vitamin B12 (pg/mL)		434.22 ± 245.41	427.85 ± 245.08	0.651

**Table 3:** Adjusted ORs (95% CI) for associations with a positive SARS-CoV-2 RT PCR test.

		Adjusted Odds Ratio		
Variable		Odds Ratio	95% Confidence Interval	*p*
Age categories	<21	1 (ref.)		
	21–40	1.566	0.64–3.77	0.917
	41–60	1.505	0.61–3.69	0.372
	≥61	1.042	0.33–3.37	0.914
Female gender		1.042	0.71–1.52	0.833
Low socioeconomic status		1.56	1.19–2.03	<0.001
Smoking	No	1 (ref.)		
	Current	0.51	0.35–0.73	<0.001
	Past	0.935	0.41–2.10	0.872
Weight change category after the bariatric surgery	Gain/no change	1 (ref.)		
	Lost < 50 kg	1.051	0.61–1.79	0.853
	Lost ≥ 50 kg	1.026	0.13–2.44	0.953
Frequency of Physical Activity	No	1 (ref.)		
	Occasionally	1.013	0.76–1.34	0.924
	1–3 times a week	1.123	0.84–1.60	0.363
	>3 times a week	0.47	0.23–0.95	0.036
Diabetes mellitus		0.949	0.69–1.29	0.742
Hypertension		1.068	0.80–1.14	0.648
Poorly controlled diabetes mellitus (Hb A1C ≥ 7%)		1.29	0.98–1.69	0.076
Vitamin D3 deficiency *		1.55	1.18–2.02	<0.001
Vitamin B12 deficiency **		1.14	0.94–1.33	0.058

* Vitamin D3 level < 20 ng/mL (75 nmol/L); ** Vitamin B12 level < 200 pg/mL.

## Data Availability

All statistical analyses are available upon requests. Because of ethical and privacy issues, the patients’ data cannot be shared.
